# Supplementary material for: Gynomonoecy in a mycoheterotrophic orchid Eulophia zollingeri with autonomous selfing hermaphroditic flowers and putatively outcrossing female flowers
Source: PeerJ. 2020 Oct 27;8:e10272. doi: 10.7717/peerj.10272 (PMC7597633; doi:10.7717/peerj.10272)
Supplement: Supplemental Information 5 — (A) Column with a degenerate rostellum, which facilitates autogamy. (B) Column with neither a rostellum nor anther cap and pollinaria. AC, anther cap; PO, pollinaria; ST, stigma. [file peerj-08-10272-s005.pdf]

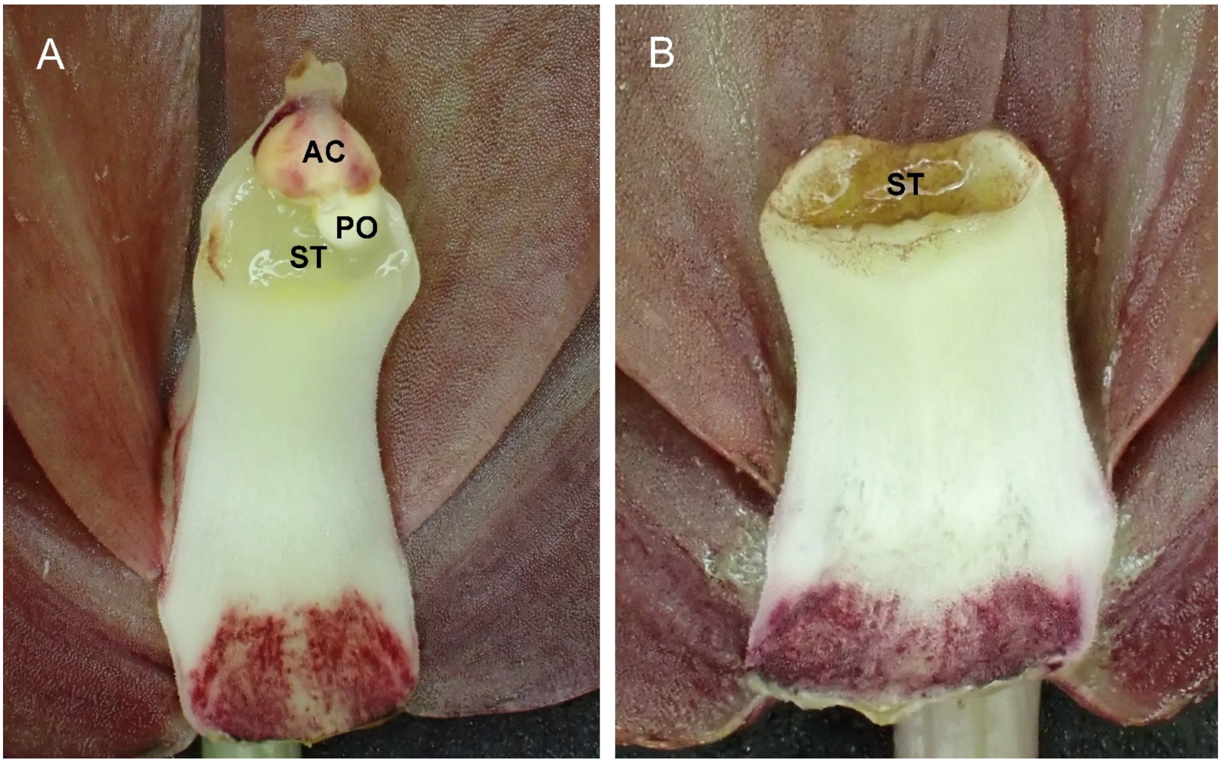

Figure S1 Column morphology of *Eulophia zollingeri* flowers in Ogimi-son, Okinawa, Japan. (A) Column with a degenerate rostellum, which facilitates autogamy. (B) Column with neither a rostellum nor anther cap and pollinaria. AC, anther cap; PO, pollinaria; ST, stigma.
